# Compartmental immunity in intra-abdominal infection: from peritoneal defense to systemic sepsis

**DOI:** 10.3389/fimmu.2026.1827984

**Published:** 2026-04-27

**Authors:** Xiao Wang, Xinping Yu, Zhenglin Chen, Yanbo Chang, Tao Ma

**Affiliations:** 1 Department of General Surgery, Tianjin Medical University General Hospital, Tianjin, China; 2 Tianjin General Surgery Institute, Tianjin Medical University General Hospital, Tianjin, China; 3 Department of Integrative Chinese and Western Medicine, Tianjin Medical University General Hospital, Tianjin, China; 4 Tianjin Key Laboratory of Precise Vascular Reconstruction and Organ Function Repair, Tianjin, China

**Keywords:** B1 cells, compartmental immunity, innate lymphoid cells, intra-abdominal infection, intra-abdominal sepsis, milky spots, peritoneal resident macrophages

## Abstract

Complicated intra-abdominal infection (cIAI) represents a common and challenging surgical emergency that frequently progresses from localized infection to intra-abdominal sepsis (IAS), leading to rapid clinical deterioration, early organ dysfunction, and unfavorable outcomes. However, the immunological mechanisms underlying these clinical behaviors remain incompletely understood. This review advances a compartment-oriented immunopathological framework to explain the unique behavior of abdominal infection across the cIAI–IAS spectrum. The peritoneal cavity is not an immunologically passive space but a highly specialized immune compartment pre-equipped with fat-associated lymphoid clusters (milky spots), peritoneal resident macrophages (PRMs), B1 cells, and innate lymphoid cells (ILCs). Upon intra-abdominal contamination, this regional immune network enables rapid and high-intensity local inflammatory responses that initially favor containment of polymicrobial infection. However, inadequate or delayed multimodal intervention, together with unfavorable host conditions such as advanced age, immunosuppression, comorbidities, and high disease severity, may permit excessive inflammatory amplification and peritoneal barrier failure within this confined anatomical space. As a consequence, pathogen- and injury-associated signals disseminate rapidly through vascular and lymphatic pathways, driving progression from cIAI to IAS. Together, this compartment-oriented perspective challenges the traditionally source control–focused understanding of cIAI by highlighting the critical role of peritoneal immune compartmentalization, opening avenues for earlier risk stratification and immunologically informed, stratified intervention strategies across the full cIAI–IAS spectrum.

## 1 Introduction

Intra-abdominal infections (IAIs) encompass infectious processes involving the peritoneal cavity and abdominal organs and are conventionally classified as uncomplicated or complicated intra-abdominal infection (cIAI) according to whether the infection is confined to the primary organ. cIAI are defined by breach of the anatomical barrier of the primary organ with extension into the peritoneal cavity, resulting in localized or diffuse peritonitis and/or intra-abdominal abscess formation (1). cIAI is one of the most common and challenging emergencies encountered in general and emergency surgery. Characterized by abrupt onset, polymicrobial burden, and intense inflammatory responses, cIAI frequently require urgent surgical or interventional source control combined with antimicrobial therapy (1, 2). Nevertheless, in clinical practice, a considerable proportion of patients experience abrupt progression to intra-abdominal sepsis (IAS), frequently accompanied by early organ dysfunction and poor outcomes. Epidemiological studies indicate that cIAI represents one of the leading infectious sources of sepsis in clinical practice, accounting for approximately 18–22% of all sepsis cases worldwide (3, 4). Notably, once cIAI progresses to IAS, the disease enters an advanced and particularly malignant phase of sepsis, characterized by highly heterogeneous clinical trajectories, early organ dysfunction, and poor outcomes (5, 6).

This clinical predicament, characterized by rapid disease progression, high severity, and poor outcomes across cIAI and its progression to IAS, is closely linked to the unique regional immune characteristics of the peritoneal cavity. The peritoneal cavity harbors a specialized regional immune system composed of peritoneal resident macrophages (PRMs), B1 cells, innate lymphoid cells (ILCs), and non-classical lymphoid structures such as fat-associated lymphoid clusters (FALCs), typified by milky spots (7, 8). These immune components are strategically positioned in a state of heightened readiness and can be rapidly activated following intra-abdominal contamination, enabling prompt local immune responses aimed at early pathogen containment (7, 9, 10). However, once the local immune barrier is breached, this exaggerated local peritoneal response is prone to spill over into the bloodstream, driving systemic inflammatory-immune imbalance and ultimately promoting the development of IAS (9, 10). Despite these insights, how compartment-specific peritoneal immune responses are dynamically regulated, and how their dysregulation, manifested as exaggerated, sustained, or non-resolving regional immune activation, drives disease escalation and subsequent clinical deterioration, remain incompletely understood. Addressing these knowledge gaps is of substantial clinical relevance. A deeper understanding of peritoneal compartment–specific immune dynamics could provide an immunologically grounded framework to support earlier risk stratification and more precise, stratified management strategies, integrated with current multimodal infection management paradigms across the full spectrum of cIAI and IAS.

## 2 IAI, cIAI and IAS

According to the consensus jointly issued by the Global Alliance for Infections in Surgery (GAIS) and the World Society of Emergency Surgery (WSES), IAIs are classified as uncomplicated or cIAI based on whether the infection remains confined to the primary organ (1). Epidemiological studies indicate that cIAI constitutes the second most common infectious source of sepsis worldwide, surpassed only by pulmonary infections, underscoring its substantial contribution to the global burden of sepsis (3, 4).

cIAI represents one of the most common and clinically challenging acute abdominal emergencies, most frequently arising from gastrointestinal perforation, anastomotic leakage, ischemic or necrotic bowel, complicated appendicitis or diverticulitis, biliary tract infection, and postoperative intra-abdominal infections. Clinically, cIAI typically presents with acute abdominal pain, signs of peritoneal irritation, systemic inflammatory responses, and varying degrees of organ dysfunction, reflecting its abrupt onset and rapid progression. Management typically requires an integrated strategy encompassing timely surgical source control, early empiric broad-spectrum antimicrobial therapy, and organ-supportive care. Nevertheless, a substantial proportion of patients continue to deteriorate and ultimately progress to intra-abdominal sepsis (IAS), likely owing to the combined influence of microbial burden, host immune status, underlying comorbidities, and delayed or insufficient therapeutic intervention (1, 2, 11).

Once cIAI evolves into IAS, patients frequently develop hemodynamic instability, elevated lactate levels, and multiple organ dysfunction, accompanied by a marked worsening of prognosis. Data from the WISS (WSES cIAI Score) study demonstrated a stepwise increase in 28-day mortality with escalating sepsis severity among cIAI patients, reaching nearly 70% in those with septic shock (12). Similar findings were reported in a single-center study from China, in which 28-day mortality increased from 3.5% in patients without sepsis to over 30% in those progressing to septic shock (13).

Increasing evidence further suggests that the anatomical source of infection critically influences sepsis outcomes. Notably, compared with sepsis arising from other infection sources, most prominently pulmonary infections, IAS is generally associated with more rapid disease progression and higher mortality (6, 14). A retrospective analysis of more than 9,000 ICU patients demonstrated that, among patients with comparable disease severity, IAS conferred a significantly higher risk of in-hospital mortality than pulmonary sepsis (6). Similar conclusions were drawn by Pieroni et al., who identified infection source as a major determinant of sepsis prognosis, with IAS associated with the highest mortality risk across different etiologies (14). Therefore, cIAI and its progression to IAS constitute a distinctly high-risk clinical continuum, characterized by infection source–specific disease trajectories and disproportionately poor outcomes that not be fully captured by generalized sepsis paradigms. These observations underscore the need to elucidate the mechanisms that govern the initiation and evolution of cIAI and its transition from a localized infection to systemic inflammation and IAS.

## 3 Regional immune composition of the peritoneal cavity: lymphoid structures, cellular composition, and physiological functions

**Figure 1:**
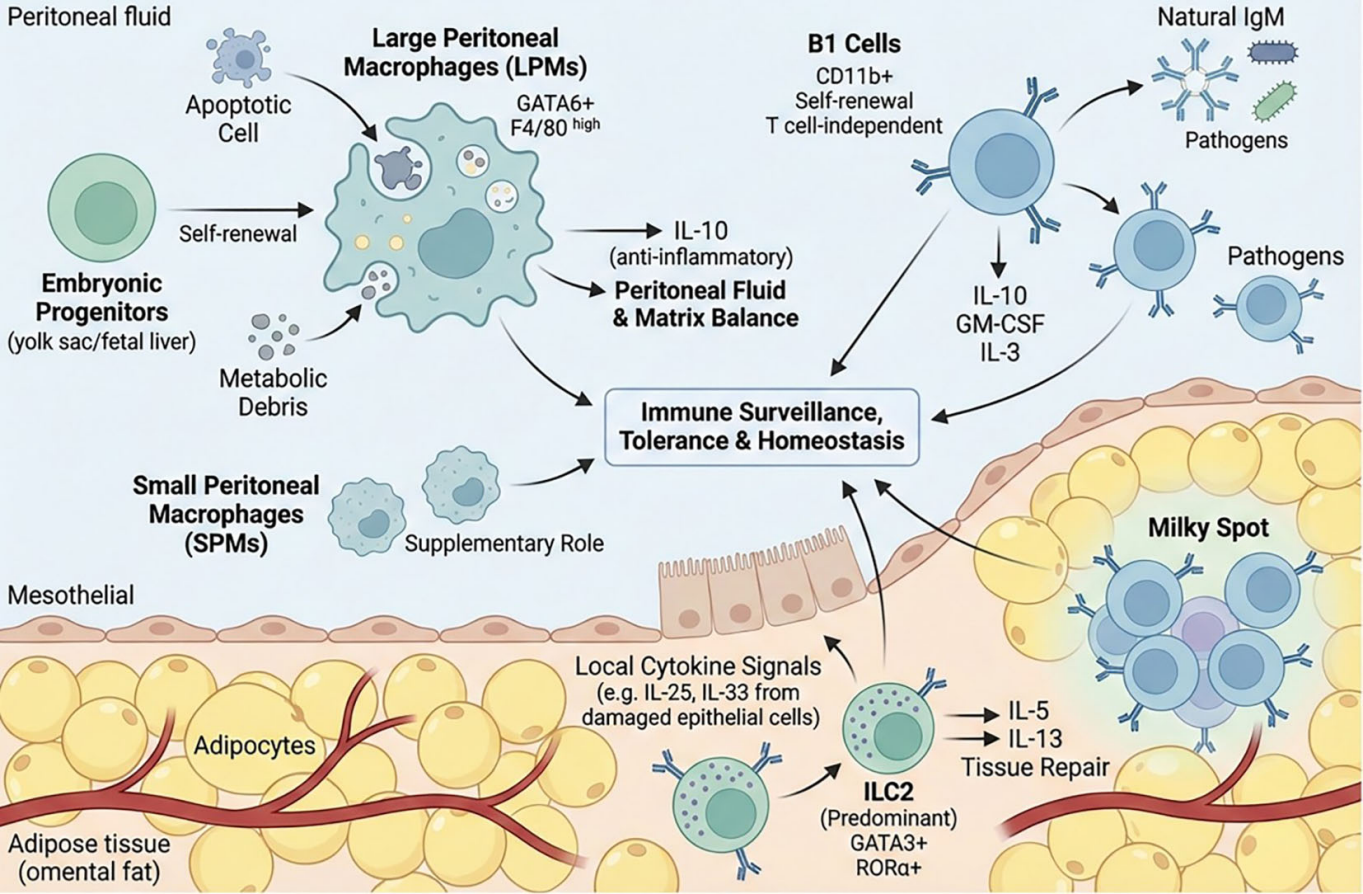
Regional immune organization of the peritoneal cavity under physiological conditions. The peritoneal cavity harbors a pre-positioned immune network characterized by milky spots, PRMs, B1 cells, and ILCs. LPMs dominate the resident macrophage pool and play central roles in immune surveillance and homeostatic regulation. B1 cells and ILC2s provide innate-like humoral and cytokine-mediated support, respectively. Together, these tissue-resident immune components maintain immune readiness, tolerance, and peritoneal homeostasis in the absence of overt infection.

The peritoneal cavity is a potential serous cavity defined by the continuity of the parietal and visceral peritoneum, lined by a monolayer of mesothelial cells covering the inner abdominal wall and the surfaces of intra-abdominal organs. Accumulating evidence from modern immunological studies has demonstrated that the peritoneal cavity is not merely an inert anatomical space, but rather a highly specialized regional immune niche composed of organized lymphoid structures and diverse immune cell populations (15). This compartment-specific immune microenvironment plays critical roles in local immune surveillance, barrier defense, and the regulation of inflammatory responses. **Figure 1** illustrates the composition and functional characteristics of the regional immune microenvironment within the peritoneal cavity under physiological conditions.

### 3.1 FALCs and milky spots: secondary lymphoid-like structures of the peritoneal cavity

FALCs represent a class of non-encapsulated, atypical secondary lymphoid–like structures capable of rapidly initiating local immune responses and are widely distributed within adipose tissues associated with body cavities. They lack well-defined cortical–medullary organization and fibrous capsules but exhibit remarkable plasticity and responsiveness under conditions of infection, inflammation, or tissue injury. Within the peritoneal cavity, milky spots constitute the most characteristic and representative form of FALCs and are particularly abundant in the omentum, mesentery, and peritoneal adipose tissues. First recognized in the nineteenth century, milky spots are whitish immune cell aggregates within omental adipose tissue (16).

**Figure 2:**
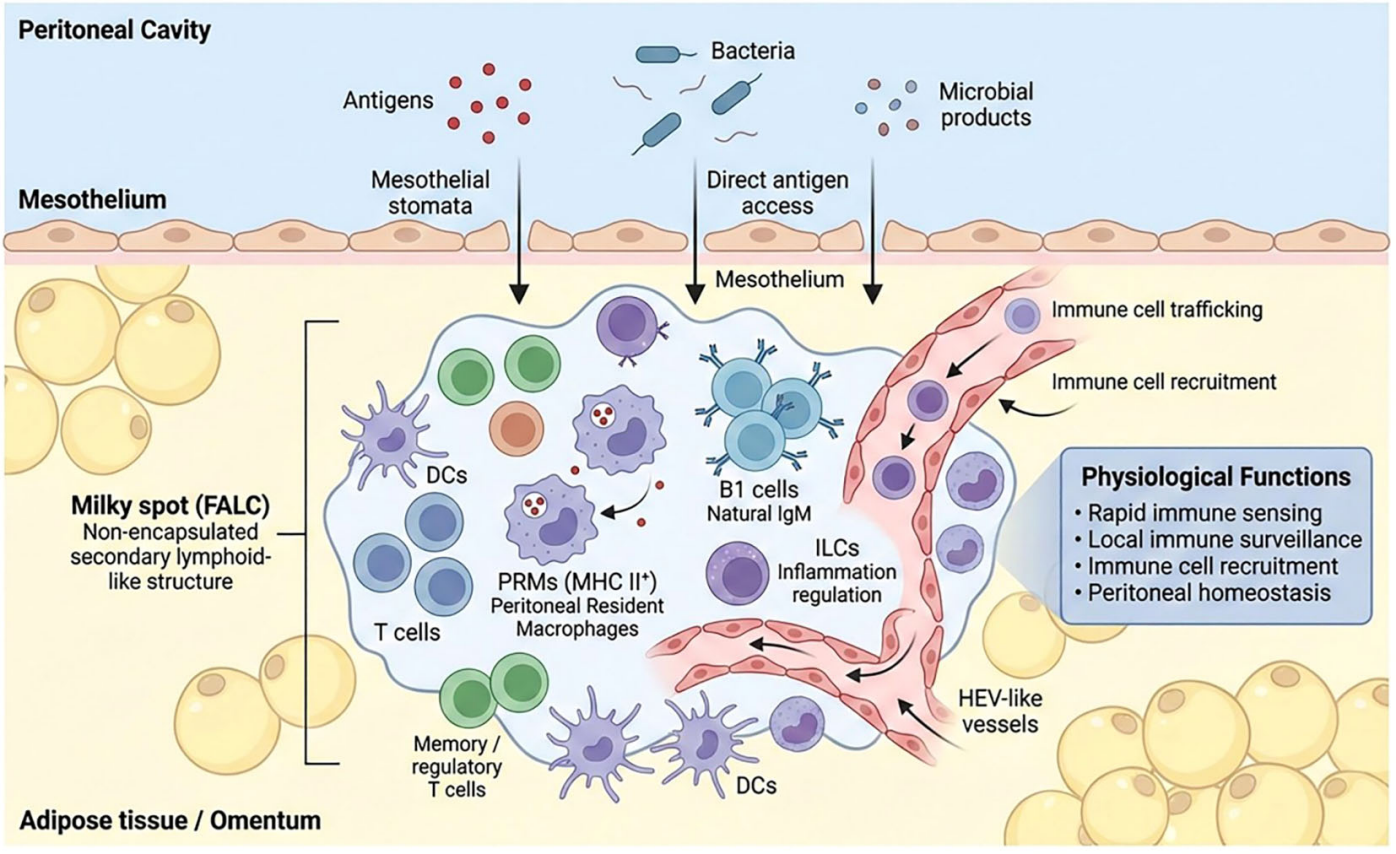
Structural organization and physiological functions of milky spots in the peritoneal cavity. Milky spots are non-encapsulated FALCs enriched in omental and peritoneal adipose tissues. Lacking classical cortical–medullary organization, they comprise diverse innate and adaptive immune cell populations, including PRMs, B1 cells, ILCs, T cells, and dendritic cells. Specialized mesothelial stomata enable direct access of peritoneal antigens and microbial products, while dense HEV-like vasculature supports immune cell recruitment and trafficking. Through this open architecture and specialized microenvironment, milky spots function as highly sensitive immune-sensing hubs that integrate local surveillance, immune activation, and peritoneal homeostasis under physiological conditions.

Milky spots are composed of a diverse array of innate and adaptive immune cells, including MHC class II–expressing PRMs, natural antibody–producing B1 cells, inflammation-modulating ILCs, as well as T cells—predominantly memory and regulatory subsets—and dendritic cells (17). Moreover, specialized mesothelial stomata-like openings between mesothelial cells overlying milky spots permit direct access of peritoneal antigens, bacteria, microbial products, and toxins into these immune structures, thereby conferring a highly sensitive immune-sensing capability (7). Upon antigen or microbial entry into the peritoneal cavity, milky spots are rapidly activated to capture antigens, initiate innate immune responses, recruit circulating immune cells, and facilitate the production of natural antibodies. In addition to their specialized cellular composition, milky spots are characterized by a dense HEV-like vascular network that serves as an efficient conduit for immune cell recruitment and trafficking, thereby enabling the rapid amplification of local immune responses (18). Collectively, through their open architecture, unique cellular composition, and specialized microenvironment, milky spots serve as highly specialized regional immune structures within the peritoneal cavity, functioning as critical hubs for peritoneal immune surveillance, homeostasis, and the monitoring of microbial invasion (10). **Figure 2** illustrates the composition and functional characteristics of the milky spot under physiological conditions.

### 3.2 Regional immune cells of the peritoneal cavity

PRMs constitute the most abundant and functionally central innate immune cell population within the peritoneal cavity. They are commonly classified into large peritoneal macrophages (LPMs) and small peritoneal macrophages (SPMs) based on phenotypic and functional differences. At the same time, this LPM/SPM classification likely oversimplifies a more dynamic and heterogeneous compartment. Current evidence suggests that the developmental origin, functional diversity, and infection-associated transitions are more complex than previously appreciated. Under steady-state conditions, LPMs predominate and serve as the principal tissue-resident macrophage population, playing key roles in maintaining peritoneal immune homeostasis. The unique microenvironment of the peritoneal cavity, shaped by adipose tissue–derived signals, mesothelial–stromal interactions, and immune structures such as milky spots, critically influences the functional identity of PRMs, distinguishing them from macrophages in other tissues. Under physiological conditions, PRMs contribute to the clearance of apoptotic cells and metabolic debris, maintenance of peritoneal fluid balance, and local immune surveillance. In addition, LPMs produce immunoregulatory mediators, such as interleukin-10 (IL-10), which constrain excessive inflammatory activation and help preserve regional immune tolerance (19–21). In contrast, SPMs are present at relatively low frequencies under steady-state conditions and are generally considered to play supplementary roles in immune monitoring and modulation, although their precise physiological functions remain incompletely defined (21, 22).

B1 cells represent a non-conventional subset of B lymphocytes, distinct from classical B2 cells, that integrate rapid innate immune–like responses with selected adaptive immune features and serve as an important component of the regional immune barrier of the peritoneal cavity (23). The peritoneal cavity represents a major enriched anatomical site for B1 cells, where they account for a substantial proportion of the total B-cell population (24, 25). Phenotypically, B1 cells are characterized by high expression of CD11b and CD43, along with relatively low expression of MHC class II and IgD, reflecting a persistently “semi-activated” state. In addition, B1 cells possess a pronounced capacity for self-renewal and rely on chemokine axes such as CXCL13 to maintain their long-term homeostatic distribution within the peritoneal cavity (25, 26). Functionally, unlike B2 cells, which require antigen-specific stimulation and T-cell help, B1 cells can rapidly respond to microbe-associated molecular patterns or endogenous danger signals in a T cell–independent manner. Upon activation, they promptly produce low-affinity but broadly reactive natural IgM antibodies, which serve as the first line of humoral immune defense against invading pathogens and mediate neutralization of microbial antigens, enhance phagocytosis, and facilitating the clearance of apoptotic cells. In addition to antibody production, B1 cells secrete multiple immunoregulatory mediators, including interleukin-10 (IL-10), granulocyte–macrophage colony-stimulating factor (GM-CSF), and interleukin-3 (IL-3), thereby contributing to the regulation of inflammatory responses and the maintenance of immune homeostasis within the local microenvironment (25).

ILCs are lymphocyte-like innate immune cells that lack antigen-specific receptors, such as T-cell receptors (TCRs) and B-cell receptors (BCRs). They are predominantly represented in the peritoneal cavity and function as tissue-resident “fixed sentinels,” exhibiting a high degree of local residency and rapid responsiveness (7, 27). Owing to their pronounced sensitivity to the local inflammatory microenvironment, ILCs differ fundamentally from conventional lymphocytes that depend on antigen presentation for activation. Instead, they can respond directly to local cytokine signals released during the early stages of infection or tissue injury, enabling prompt initiation of immune responses without the need for antigen presentation (27, 28). Among the major ILC subsets, ILC2s constitute the predominant population in the peritoneal cavity. They primarily produce interleukin-5 (IL-5) and interleukin-13 (IL-13), thereby contributing to the regulation of local inflammation, immune cell recruitment, and tissue repair processes (29). By contrast, other ILC subsets, including ILC1s and ILC3s, appear to be less abundant and remain far less well characterized in the peritoneal cavity. Nevertheless, available evidence suggests that these populations may also contribute to inflammatory regulation, cytokine production, and host defense. However, their subset-specific roles, context-dependent regulation, and interactions with other regional immune cells within the peritoneal cavity remain insufficiently defined and warrant further investigation.

At steady state, the peritoneal cavity also contains smaller populations of T cells, dendritic cells, and natural killer cells, which collectively contribute to immune surveillance, antigen sampling, and the maintenance of local immune homeostasis (7). In contrast, neutrophils and monocytes are scarcely detectable under physiological conditions. Upon infection or tissue injury, these cells are rapidly recruited into the peritoneal space, where they function as emergency effector cells, mediating pathogen clearance and amplifying early inflammatory responses during host defense (7, 9).

## 4 Compartment-specific orchestration of peritoneal immune responses upon infectious insults

**Figure 3:**
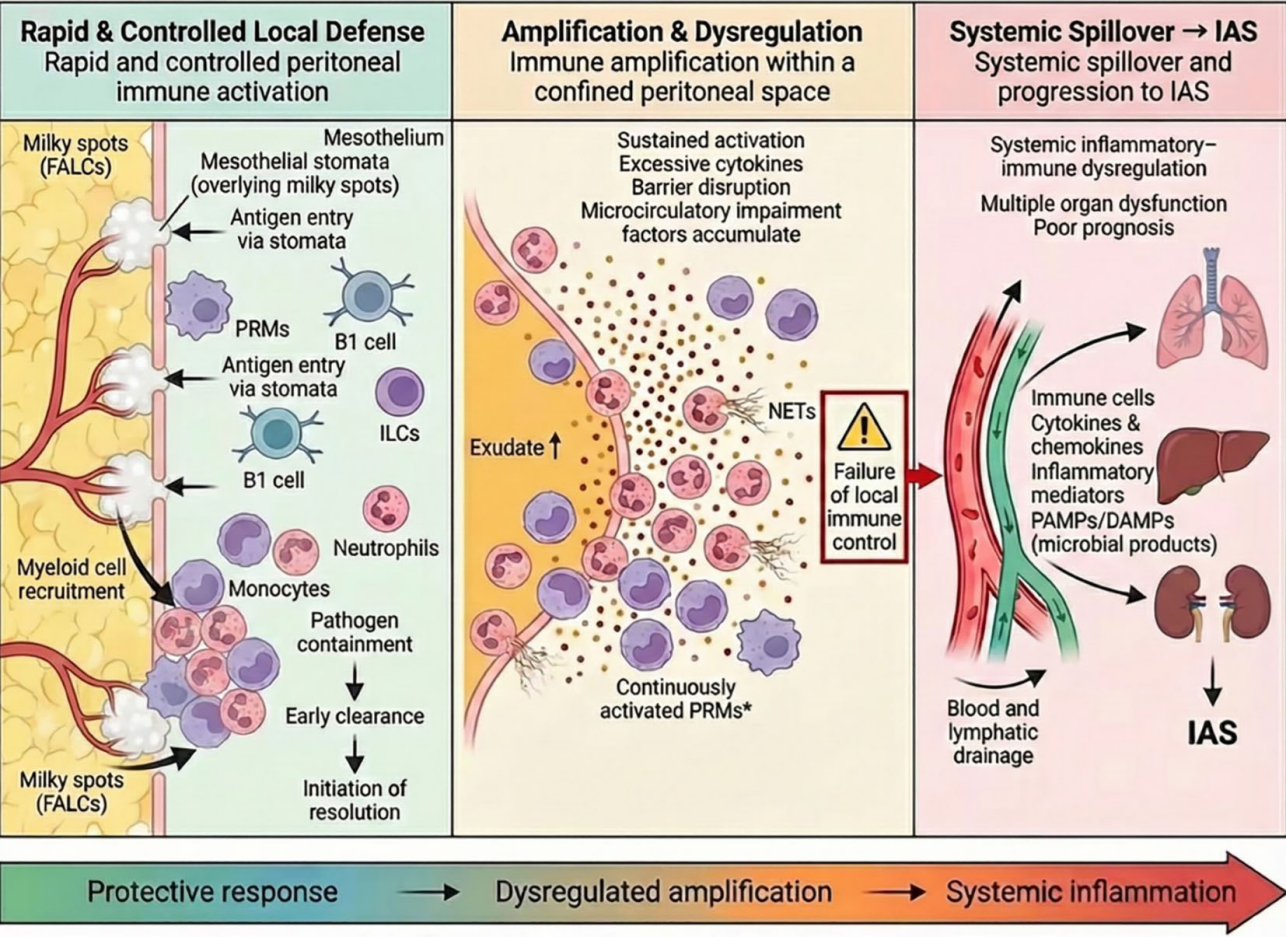
Conceptual model illustrating post-infection peritoneal immune response and its role in driving cIAI progression to IAS. The peritoneal cavity contains pre-positioned immune units that enable rapid local immune activation upon pathogen or danger signal exposure. When effectively regulated, this response promotes pathogen containment and resolution. However, sustained immune activation within the confined peritoneal space leads to excessive inflammatory amplification and barrier disruption. Through extensive vascular and HEV-like connections, immune cells and inflammatory mediators spill over into the systemic circulation, driving IAS and poor clinical outcomes.

It is increasingly recognized that immune responses are organized in a compartment-specific manner, exhibiting distinct characteristics across different anatomical sites. Such compartment-specific immunity arises from differences in anatomical architecture, immune cell composition, and local microenvironment among individual organs or anatomical compartments (30). The peritoneal cavity represents a prototypical example of a highly specialized immune compartment, in which unique immune structures, most notably milky spots, together with regionally enriched, tissue-resident innate immune cell populations, including PRMs, B1 cells, and ILC2s. Collectively, these elements establish a distinctive compartment-specific immune landscape that enables the peritoneal cavity to mount rapid, sensitive, and high-intensity immune responses to infectious or injurious stimuli (31). The mechanistic framework underlying the post-infection immune response and its role in driving the progression from cIAI to IAS are illustrated in **Figure 3**.

### 4.1 Milky spots as dynamic immune hubs in infection-induced peritoneal immune remodeling

Milky spots, a peritoneal-enriched form of FALCs, serve as one of the earliest immune structures to initiate local immune responses following intra-abdominal infection and undergo pronounced dynamic remodeling during this process. Clinically, this early activation corresponds to the rapid onset of peritoneal inflammation that characterizes the initial stage of complicated intra-abdominal infection (cIAI). Morphologically, infection is accompanied by a rapid increase in both the number and size of milky spots, manifested as enlarged and more numerous whitish nodules on the omentum, reflecting intense local immune activation (8). Within activated milky spots, PRMs are rapidly activated and execute key innate effector functions, including pathogen phagocytosis, antigen capture, and the production of cytokines and inflammatory mediators. In parallel, B1 cells residing in milky spots promptly secrete natural IgM antibodies in a T cell–independent manner, thereby contributing to early innate-like humoral protection during the initial phase of infection (32). ILCs also respond rapidly in an antigen-independent fashion, primarily exerting their effects through the secretion of immunomodulatory cytokines.

Together, PRMs, B1 cells, and ILCs within milky spots form a tightly coordinated local immune network that orchestrates early inflammatory responses and downstream immune cell cascades. Through the production of cytokines, inflammatory mediators, and chemokines, this network not only amplifies local immune activation but also governs the recruitment of additional effector cells (17). Concurrently, the HEV-like structures within milky spots facilitate the rapid influx of circulating neutrophils and monocytes, linking early regional immune activation to subsequent escalation of inflammatory cell infiltration. From a clinical perspective, milky spots therefore function not merely as passive immune aggregates, but as dynamic immunological hubs that play a pivotal role in shaping the magnitude and trajectory of inflammatory–immune responses upon infectious challenge (8, 17).

### 4.2 Infection-driven remodeling and functional reprogramming of peritoneal immune cells

Upon the onset of infection, PRMs act as the first-line sentinels within the peritoneal cavity and undergo profound quantitative and functional remodeling. By phagocytosing invading bacteria and releasing chemokines such as CCL2 and CXCL1, together with pro-inflammatory cytokines including TNF-α, IL-6, and IL-1β, PRMs initiate local inflammatory responses and promote the recruitment of neutrophils and monocytes to the peritoneal cavity for early pathogen clearance (9, 19). During this acute phase, the population of LPMs typically declines markedly. This reduction results from apoptosis or pyroptosis induced by bacterial virulence factors and inflammatory stress, as well as from the physical detachment of LPMs into the peritoneal fluid, where they are eliminated or relocate to milky spots (9, 19). Concurrently, large numbers of bone marrow–derived Ly6Chi monocytes are rapidly recruited into the peritoneal cavity and differentiate into SPMs, partially replenishing the local macrophage pool and sustaining inflammatory responses (22). However, the precise developmental and functional relationships among resident LPMs, recruited monocyte-derived cells, and SPM-like populations during infection-induced remodeling remain an area of ongoing debate. Beyond numerical alterations, PRMs exhibit pronounced functional plasticity along the disease course. During the early inflammatory phase, PRMs preferentially adopt a pro-inflammatory phenotype (commonly referred to as an M1-like state), characterized by enhanced phagocytic capacity and robust production of inflammatory cytokines that facilitate rapid pathogen clearance. As infection becomes controlled, PRMs gradually transition toward a pro-resolving and tissue-repair phenotype (M2-like state), promoting debris clearance, attenuation of inflammatory signaling, and restoration of peritoneal tissue integrity (33). Experimental evidence indicates that disruption of PRM integrity or loss of this functional plasticity is closely associated with defective bacterial clearance, prolonged inflammatory activation, and increased susceptibility to multiple organ dysfunction. Conversely, preservation of PRM homeostatic regulation appears critical for determining whether IAI resolves locally or progresses toward systemic deterioration (34).

B1 cells exert multifaceted protective functions in host defense during IAI, particularly in shaping the balance between pathogen containment and inflammatory escalation. Upon exposure to invading pathogens or danger-associated signals, B1 cells rapidly expand and secrete large amounts of natural IgM. These antibodies, characterized by low affinity but high avidity and broad polyreactivity, recognize conserved microbial structures and endogenous danger signals without prior antigen exposure. As a pre-existing component of the humoral immune repertoire, natural IgM provides immediate immune coverage during the earliest phase of infection. This early IgM response promotes pathogen opsonization, complement activation, and phagocytic clearance, thereby constituting a first-line humoral defense. Under sustained infectious conditions, a subset of B1 cells further differentiates into innate response activator B cells (IRA-B cells), which are characterized by robust secretion of GM-CSF, IL-3, and related hematopoietic cytokines. Through these mediators, IRA-B cells regulate myeloid cell development, recruitment, and antimicrobial effector functions of myeloid cells, reinforcing early host defense (35). Importantly, B1 cells also exert important immunoregulatory effects by producing interleukin-10 (IL-10), thereby restraining excessive inflammatory responses and contributing to the maintenance of immune homeostasis. Experimental studies have demonstrated that mice deficient in B1 cells are prone to uncontrolled inflammation, pronounced bacteremia, and significantly elevated mortality following intra-abdominal infection (36). Conversely, adoptive transfer of B1 cells markedly improves survival in murine models of sepsis, largely through IL-10–mediated attenuation of cytokine storm, attenuation of tissue injury, and protection of distant organs such as the lungs (36, 37). These findings indicate B1 cells as key modulators at the interface between early protection and inflammatory overactivation.

ILCs, as tissue-resident immune populations, contribute to the fine-tuning of peritoneal immune responses following infectious challenges. Unlike adaptive lymphocytes, ILCs do not require antigen presentation and instead respond directly to local stress- and inflammation-associated cytokines, including IL-25, IL-33, TSLP, and IL-12, which are released early during infection or tissue injury (28). Among ILC subsets, ILC2s are currently the most extensively studied and functionally prominent population in the peritoneal cavity. Upon activation, ILC2s produce type 2 cytokines, particularly IL-5 and IL-13, which orchestrate multiple downstream processes relevant to disease trajectory. These include modulation of neutrophil responses, enhancement of natural IgM production by B1 cells, recruitment of eosinophils, and promotion of macrophage polarization toward a tissue-repair–associated phenotype. Through these coordinated actions, ILC2s contributes to balancing antimicrobial defense with inflammation resolution and tissue repair within the peritoneal cavity (38). Notably, dynamic changes in ILC2 populations have been observed during infection progression, with expansion in the peritoneal cavity and migration of activated subsets to distant organs such as the lungs and liver. At these sites, ILC2s participate in systemic immune modulation and tissue protection, underscoring their role as a functional link between regional peritoneal immunity and systemic inflammatory responses (39). Dysregulation of this regulatory axis may therefore favor persistent inflammation and contribute to adverse systemic outcomes. Despite the current emphasis on ILC2s, the potential contributions of ILC1 and ILC3 populations to peritoneal inflammatory regulation and antimicrobial responses remain underexplored.

Although neutrophils and monocytes are scarcely present in the peritoneal cavity under homeostatic conditions, infection rapidly induces emergency myelopoiesis and bone marrow mobilization, leading to a massive influx of these cells into the peritoneal space. Clinically, this recruitment wave corresponds to the acute inflammatory escalation phase of intra-abdominal infection (IAI) and represents a hallmark of peritoneal immune activation that is essential for early pathogen containment (40, 41). Neutrophils constitute the predominant early effector population and exert potent antimicrobial activity through phagocytosis, oxidative burst, degranulation, and neutrophil extracellular trap (NET) formation, thereby effectively reducing bacterial burden and limiting pathogen dissemination (40). Beyond microbial killing, neutrophils amplify local inflammation by releasing proteases, cytokines, and chemokines, which further enhance immune cell recruitment and activation. However, excessive or prolonged neutrophil activation can aggravate tissue injury and drive inflammatory pathology, highlighting their dual role in host defense and immunopathology (42). In parallel, recruited bone marrow–derived monocytes exhibit marked functional plasticity within the infected peritoneal environment. After infiltration, monocytes differentiate into macrophages or dendritic cells, contributing to pathogen clearance, antigen presentation, and inflammatory amplification. As infection becomes controlled, subsets of monocyte-derived cells acquire pro-resolving and reparative phenotypes, characterized by enhanced efferocytosis, secretion of anti-inflammatory mediators, and promotion of tissue repair. Through this dynamic transition, monocytes serve as a critical link between effective pathogen clearance and the resolution of inflammation and restoration of tissue homeostasis (41).

### 4.3 Peritoneal compartment–driven immune escalation, clinical heterogeneity, and translational implications in cIAI and IAS

As discussed above, the peritoneal cavity is pre-equipped with FALCs, typified by milky spots, together with regionally enriched innate immune cell populations. These immune structures and cell populations remain in a heightened state of immune readiness and can be activated almost instantaneously upon pathogen exposure, enabling a rapid and robust local inflammatory response from the very onset of infection (7, 9). Clinically, this immune configuration underlies the frequently observed “high starting point” of inflammation in cIAI, in which patients may already present with marked inflammatory responses at initial surgical or intensive care assessment.

Functionally, the peritoneal immune response is orchestrated through a relay-like interaction between upstream regulatory immune units and downstream effector cells. Milky spots act as central immune hubs that integrate antigen capture, immune cell activation, and leukocyte recruitment, while PRMs, B1 cells, and ILCs shape the magnitude and timing of local immune activation (9, 17). Under this regulatory framework, neutrophils and monocyte-derived cells are rapidly recruited as dominant effector populations responsible for microbial killing and inflammatory amplification. This hierarchical organization helps explain why, once the inflammatory response is triggered, peritoneal infection tends to progress in a self-amplifying manner and becomes difficult to reverse if early control is not achieved.

Such rapid and robust immune activation confers important pathophysiological advantages. IAIs most commonly arise from disruption of the gastrointestinal barrier and are typically associated with complex polymicrobial communities, including facultative anaerobes, strict anaerobes, and both Gram-negative and Gram-positive bacteria. These events are accompanied by the release of multiple danger signals, including endotoxins, microbial fragments, and necrotic tissue–derived components, collectively providing potent inflammatory stimuli. Under these conditions, prompt amplification of local inflammatory responses facilitates early containment and clearance of invading pathogens, thereby limiting bacterial dissemination, and supporting the subsequent resolution of inflammation and tissue repair (1, 7, 9). The ability to mount such a timely and effective regional immune response is critical for achieving effective infection control and for reducing the risk of subsequent disease progression and clinical deterioration.

However, the same rapid and intense peritoneal inflammatory-immune activation may also predispose to maladaptive amplification and pathological injury. As a confined anatomical cavity, the peritoneal space has a limited capacity for spontaneous clearance of inflammatory exudates, necrotic debris, and purulent contents following infection. When coupled with increased intra-abdominal pressure and microcirculatory hypoperfusion, this impaired clearance promotes local tissue ischemia and prolongs the inflammatory burden within the peritoneal compartment. Consequently, when infection is not effectively controlled, a persistently pro-inflammatory microenvironment is established, reinforcing a self-perpetuating vicious cycle of immune activation, tissue injury, and impaired inflammatory resolution (43, 44). At the same time, the peritoneal cavity is characterized by dense vascular and lymphatic networks that provide close anatomical and functional connectivity with the systemic circulation. Once the local immune barrier is breached, pathogens, toxins, pro-inflammatory cytokines, and other inflammatory mediators can rapidly disseminate into the bloodstream, triggering systemic inflammatory cascades and immune dysregulation, thereby driving the progression from cIAI to IAS. Clinically, this provides a biological framework for understanding why some patients continue to deteriorate despite technically adequate source control, as inflammatory escalation may already have transitioned beyond local containment.

When intervention is achieved within the early and relatively controllable window of immune activation, timely and adequate source control combined with early and appropriate antimicrobial therapy can often confine inflammatory amplification to the peritoneal compartment, thereby allowing effective disease control (2, 11). In contrast, delayed or insufficient source control permits continued escalation of peritoneal immune activation within the confined anatomical space, driving a self-amplifying inflammatory process and substantially increasing the risk of regional inflammation spilling over into the systemic circulation. Importantly, clinical practice indicates that a considerable proportion of patients with cIAI may continue to deteriorate despite technically adequate surgical intervention and antimicrobial therapy. This unfavorable trajectory likely reflects not only residual infectious burden, but also the influence of host-related factors. Advanced age, immunosuppression, major comorbidities, and high baseline disease severity may compromise peritoneal compartmental immune homeostasis to varying degrees by weakening early local containment, altering macrophage and lymphoid cell responsiveness, impairing inflammatory resolution, and lowering the threshold for transition from regional immune dysregulation to systemic deterioration. Together, these factors may enable regional immune responses to surpass local regulatory thresholds and evolve into systemic inflammatory–immune dysregulation, ultimately culminating in IAS (5, 6, 11). Integrating such host-dependent modifiers into the compartment-specific framework is essential for understanding why patients with apparently similar abdominal infections may follow markedly different clinical course.

A deeper understanding of peritoneal regional immune responses carries important clinical and translational implications, particularly for the identification of early biomarkers, patient stratification, and the development of mechanism-informed therapeutic strategies (45, 46). There is a clear need to identify biomarkers that reflect dynamic transitions in peritoneal immune status, thereby supporting early risk stratification and recognition of patients at risk of deterioration (45, 46). In parallel, beyond the classical principles of source control and antimicrobial therapy, it remains essential to define the key regulatory nodes governing peritoneal regional immune imbalance (2, 11, 47). Such insights may provide a foundation for precision immunomodulatory strategies aimed at interrupting pathological immune amplification and preventing progression toward systemic inflammation. From a translational perspective, although therapeutic strategies directly targeting peritoneal compartmental immunity remain largely at the preclinical stage, several lines of investigation suggest potential intervention points. These include modulation of PRM functional polarization to preserve antimicrobial defense while limiting maladaptive inflammatory amplification, enhancement of protective B-cell–associated responses such as natural IgM- and IL-10-related pathways, and approaches aimed at preserving or restoring the immune-organizing functions of milky spots and related peritoneal lymphoid structures (10, 35–37). Although such strategies are not yet ready for routine clinical application, they help place the present conceptual framework in a more actionable translational context.

In addition, a better understanding of these characteristics of the peritoneal compartment may also help explain why IAS secondary to peritonitis follows a pathophysiological trajectory distinct from that of sepsis arising from pulmonary and other common infectious sources. Compared with pulmonary infection, the most common source of sepsis, peritonitis develops not within a continuously ventilated mucosal organ equipped with epithelial clearance mechanisms, but within a confined serous cavity characterized by limited intrinsic drainage and a pre-positioned regional immune network centered on milky spots/FALCs, PRMs, B1 cells, and ILCs (6, 7, 10). Accordingly, once intra-abdominal contamination occurs, the peritoneal cavity is exposed not only to polymicrobial invasion, but also frequently to digestive fluid leakage, fecal material, necrotic tissue, and a substantial burden of pathogen- and danger-associated signals, which together may trigger rapid and high-intensity local inflammatory amplification. In contrast to the lung, where local host defense is organized around the alveolar–epithelial barrier and continuous airway clearance, the infected peritoneal cavity has limited capacity to remove accumulating purulent exudates and inflammatory debris, particularly in the setting of increased intra-abdominal pressure and microcirculatory dysfunction (43, 44). Under such conditions, failure of local containment may permit rapid dissemination of microbial products and inflammatory mediators through the extensive vascular and lymphatic connections of the peritoneal compartment, thereby favoring early systemic spillover and inflammatory–immune dysregulation. Together, these features may help account for the distinctive escalation pattern and unfavorable outcomes of cIAI and IAS relative to sepsis from other infectious sources (6, 43). More broadly, these observations underscore the importance of fully appreciating the heterogeneity of sepsis, identifying the source-specific mechanisms that shape its clinical course, and developing targeted therapeutic strategies on this basis.

## 5 Conclusions and perspectives

Overall, the compartment-specific immunity of the peritoneal cavity represents a central determinant underlying the distinctive behavior of cIAI and the propensity of abdominal sepsis for rapid escalation and poor clinical outcomes. The immune architecture that enables rapid and robust local pathogen control, driven by milky spots, PRMs, B1 cells, and ILCs, also creates a setting in which immune dysregulation, once initiated, is readily amplified by the confined peritoneal anatomy and extensive vascular and lymphatic connectivity. When local immune containment and timely infection control are achieved, this regional immune response supports infection resolution. However, failure of peritoneal immune regulation transforms the abdominal cavity into a powerful amplifier of systemic inflammation, driving progression from cIAI to IAS. These insights highlight that the adverse clinical consequences associated with cIAI and IAS are not determined solely by pathogen burden or systemic inflammation, but critically by the integrity and regulation of peritoneal regional immunity.

Despite substantial advances, a comprehensive understanding of how peritoneal regional immunity dynamically evolves during cIAI and drives progression to IAS remains incomplete. Key gaps persist in delineating the temporal remodeling and functional regulation of milky spots during infection, the spatiotemporal coordination of the highly sensitive immune interaction network formed by PRMs, B1 cells, and ILC2s, and the pathophysiological mechanisms through which regional immune imbalance propagates systemic inflammation and immune dysregulation. Addressing these unresolved issues will be essential for refining the pathophysiological continuum from cIAI onset to IAS, and for providing a robust conceptual framework for informing earlier recognition, risk stratification, and mechanism-based targeted therapeutic strategies, ultimately improving outcomes in patients with cIAI and IAS.
